# The Marvel of Race Existence

**Published:** 1884-04

**Authors:** Ben. H. Pratt


### THE BISTOURY.

Vol. XX. ELMIRA, N. Y., APRIL, 1884. No. 1.

### THE MARVEL OF RACE EXISTENCE.

BEN H. PRATT.

THE WONDER HOW HUMAN MATURITY IS ACHIEVED—WHY HAS THE HUMAN FAMILY NOT BECOME EXTINCT?—A SCHOOL FOR THE TEST OF RACE POSSIBILITIES—IS DOGMA THE FOE OF PHYSICAL PERFECTION?—WHY NOT BREED MEN?

The earth has become peopled; and, if tradition and revelation are not sadly false, from a single pair. Statistics prove that human kind increases with every decade. From the beginning of the race to the present time, there is no evidence that any period, save the one of the deluge, has ever witnessed a diminution of the human family, but on the contrary, a constant increase.

Few halt in their headlong living to consider how wonderful it is that populations increase throughout the world. Despite pestilences, and famines, and floods, and earthquakes, and volcanoes, and lightnings, and cyclones, and disease, and old age, and wars, and murders, and all the varied circumstance of accident, men increase and multiply with an almost miraculous, if not appalling, rapidity.

It is not only amazing that this is so, but it is one of the incredible phenomena of vital economy that the human kind should ever have perpetuated itself beyond a few generations, at the most. It is possible to conceive how, in the rudeness of the primitive times, men, women and children, in a state of nature, with no luxurious temptations to indulgence, with a constant necessity for activity, with only the spontaneous fruits of the earth for nourishment, and the loosely donned skins of creatures for raiment, this natural man should naturally live, and grow, and multiply, and die at a marvelous old age. Disease had not come among mankind, for disease is the penalty of disobedience of law; food was absolutely wholesome; dress was neither cumbersome nor confining nor distorting; inertness was not possible, even if craved, for man was compelled to get his living, and the means of inviting death and race extinction had not been invented. It is credible that human kind should have increased under such environment until it had gained wisdom sufficient to accomplish its extinction.

Having outlived its rude and natural state; having achieved civilization, little by little; having abandoned its life-giving and life-prolonging habits; having adopted manners and customs and food and raiment and occupation and diversion not in harmony with the reproduction or prolongation of life, how is it that mankind continues? More marvelous still, how is it possible that the race grows numerically and promises to cover the whole earth with its vastly multiplying offspring?

Leaving out of the computation the destructive tendencies of the elemental convulsions of nature; eliminating from the summing up of the destructive powers the devastations that now and again befall wide portions of the earth; making no account of the millions of annual suicides by shot, and steel, and drug, and bludgeon, and rope, and close rooms, and corsets, and gluttony, and little rest, and unrest, and stimulation, and insomnia, and mind-drive, and care, and indolence, and revels, and all the varieties and indiscretions of living; leaving all these out of the catologue of race-destroying elements, and presuming that there is but one means of race extermination, to wit: juvenile and parental recklessness and the opportunities provided therefor, and it is even then a wonder among wonders that the last youth has not long since gone the way of all flesh, leaving the old to die off and depopulate the world.

But the race increases, and the young continue to live through their youth, and manage to attain maturity and even old age! It is almost incomprehensible. Why, to the ordinary observer, who knows what and how many are the deadly foes lurking in the pathway of childhood, the chances that one born into the world shall attain to even the age of discretion are seemingly as infinity to zero. To those cognizant of the fatalities of juveniledom there is no apparent hope of rearing a son or daughter of man, yet sons and daughters are, and they are hosts upon hosts. Verily, it is a miracle, this existence, this maintaining of the vital powers under such circumstances.

Why, the very birth of a human infant, in this age, is a marvel. That the delicate lump of reddish brown, shriveled and limp vitality which we call a new-born infant, should live through parturition, is a miracle in itself. Nay more, how ever in the world that mite of being endures its ante-parturitional period, and hangs on to life, is a mystery of mysteries. There is everything to quench its life, and scarcely anything to retain it. There are a hundred circumstances that would seem, in the fetal development period, to combine for the absolute extermination of vitality. The dress that maternal pride, and the desire of concealment, and the chagrin over the inevitable, and impatience of coming events, and determination to put off the recluse period as late as desperation may suggest, is murderous, to begin with. That children are born at all, is an evidence of the marvelously sustaining and resisting powers of human nature. That they should be born alive is a mystery; but that they should live an hour, days, live at all, is a wonder unfathomable by the reflecting mind.

But, the mystery of parturition having been practically solved, and its concomitant dangers having been safely passed, and while one is meditating upon the mystery that not only vitality remains, but a really vigorous exhibition of it, lo, this speck of humanity, this germ of an actual man or woman, continues not only to live, but to actually grow, and strengthen, and exhibit indications of budding intellect; and these again amid the most unpropitious, nay, absolutely opposing circumstances. The food, the dress, the dandling, the care, the entertainment, the sleep, the play, all are beset with insidious and apparently unavoidable fatalities, and some of which, it seems absolutely certain must result in closing the career of the infant of tender years.

Watch you how they feed these babies of ours. It is a continual programme of stuffing and pouring. If the child whimpers, stuff it; if it suffers from the stuffing, stuff it more; if it falls and cries, stuff it; if a concealed pin is somewhere piercing its anatomy, stop the scream with a mouth full of nipple or bottle or spoon victuals; stuff it until its little belly looks like a bleached foot-ball with a bust on it and two legs dangling from it; stuff it, nature rebelling against it all the while, and heaving up the superfluous stuffing; stuff it, though belchings and coughings and chokings prove that the gastric magazine is surcharged and ready to spontaneously burst asunder at any instant! Alas for the stuffing that makes a dyspeptic of the child before it sheds its belly-band?

Watch how they pick up and hold and "tend" the baby. Generally the nurse's grip is under the arms, lifting the shoulders up to the ears, bending the yet cartilaginous clavicles, distorting the yet plastic ribs, compressing the lungs laterally, nearly dislocating the humeri, overlapping the scapulæ, and humping the back. Then they flop it belly-bumber wise, over the knee, and jounce it on its stomach until the propulsionary effort discharges the contents of stomach and even bowels. Then they whip it over on its back, letting its heavy head dangle by its tender neck until the child's eyes almost protrude from their sockets, and it turns red with a cerebral rush of blood. Then they pick it up by the ankles, yank its body and breech about, the knee of the yanker serving as a fulcrum on which this baby must be see-sawed and rotated during the diapering process. Then, in the washing and dressing routine that baby must daily go through, there isn't a position in all the formulæ of gymnastic science that the infant is not slung and pitched and plumped and squatted and rolled and thumped into; and yet, if the baby doesn't come up smiling and chuckling after all this mauling and twisting and slinging and flopping, the nurse is afraid it is sick, and the stuffing and the jouncing are begun over again, and, wonder on wonders, the little thing not only lives through it all, but actually seems to thrive and grow and have entire muscles and straight bones and healthy organs, and developes, miraculously, into a maturing human being.

Then, as soon as its spine is strong enough to enable the child to sit up, and it has learned how to keep its center of gravity over its beam ends, it is given all the loose furniture about the house that it can lift, and it then amuses itself with sucking dirt and paint, and chewing wool and hair, and biting everything small enough to get into its mouth, and picking pins, and beads, and buttons, and dirty crumbs, and wads of paper, and feathers from the pillow, and strings, and hair pins, and broom splints, and matches, and specks of miscellany brought in from out doors on the family soles, and all these, or such portions of them as the youngster doesn't choke on and have extracted by force from its mouth, go into the stomach to help swell the bowel-case that looks so much like an untanned foot-ball. How does the baby ever live through it all?

But it keeps on growing and strengthening. By and by it climbs up against everything, and tumbles over into and upon everything, and bumps its head, and smashes its nose, and puts things into its ears and nostrils, and cuts its gums on combs and broken china, and gets its fingers pinched in door-cracks and under chair-rockers, until it waddles off on its own feet and trips over wrinkles in the carpet, or stumbles over the sills of the door-ways, or pitches headlong into the grate, or rolls down the cellar stairs, or off the porches, or plunges headlong into the wash tub, or pulls the plants from shelves and is buried amid ruins of earth and crockery and leaves; runs its arms up to the shoulders into the spittoon and chuckles with glee over the mess it finds there and daubs itself with; pulls the table-cloth from the breakfast board and screams when the china comes; pulls the pans from the sink and the skillets and griddles from the stove; sticks its fingers into the hot pudding, and wears so many rags for cuts and burns, and so many plasters for bumps and bruises, that it looks like an Egyptian mummy half unrolled; and yet the child lives through it all. Despite hammers and pinchers and rockers and high porches and hot fat and boiling pots and feeding on plaster from the hole in the wall, that child lives to be a big enough boy or girl, and to get out doors and encounter still greater dangers there.

This marvel of human toughness has passed through the ordeal of infantile disasters only to meet still more terrible ones. Even yet it seems to bear a charmed life. It slides on the cellar doors and gashes itself raw; it falls on the ice and doesn't crack its skull nor fracture more than one or two limbs; it skates on ice, and on rollers, and turns summersaults, and scares the beholder almost to death, and gets up smiling and tries it all over again; it darts with lightning speed down hill on a sled and plunges into tree-boxes, and running sleighs, and horses' heels, and comes out alive and grinning; it is switched off the tail-board of the dashing sleigh it has caught on to, and is hurled from the car it would steal a ride on; it climbs all the ladders and scales all the roofs, and gets up into all the trees, and pitches out of all the mows, and rides bare-back all the horses in the neighborhood, and goes in swimming in the deepest holes, on the hottest days, in the broiling sun; it skips rope, on the count, up into the thousands, and falls down exhausted from the over strain upon nerves and muscles, and lives through it; it does the tickly-bender dare act on ice, gets soused in over head and ears, and catches nothing more than a sound threshing at the hands of the parent as soon as the victim is dry enough to handle. It gorges itself with jam, and cookies, and mince pie, and keeps its stomach in a constant state of ferment, nature the while doing some wondrous feats for that child's physical salvation in its efforts to counteract all this defiance of dietetic law. But the boy and girl live through it all —live to grow up to tight garters and tighter corsets, and bare arms, and bare necks, and half-clad limbs, and late hours and dizzy dances, and midnight suppers, and exposure to all sorts of weather; and despite all these, and the doctor's oft demanded and oft swallowed prescriptions, and the clandestine resorts to all sorts of nostrums for the liver, and the lungs, and the complexion, and the teeth; for all this the youth keeps on in apparent bloom and cheerfulness, and reaches maturity by a process the mystery of which we all stand in mute wonder over; and it makes us shudder to think how it ever got through it all, and compels our admiration of the wonderful. the grand conservatorial powers that nature keeps latent for just such emergencies as these.

The battle between circumstance and nature's preservative powers is constant in childhood, and in youth the conflict is none the less hotly waged between indiscretions and recklessness on the one side, and the power of human vitality on the other. Talk about the wonderful discoveries of the age! Of the startling inventions of the time! Of steam's achievements, of electricity's revelations, of science's demonstrations! They are but trifles compared with the old, old wonder as to how in the world nature manages to prevent the extinction of the civilized portion of the human family before it reaches the age of discretion.

One would think that among all the schools of philosophy that are arising and laboring in the field of reform of all sorts, there would have arisen a school whose object should be the physical bettering of the human race by a return to primitive and wholesome means of begetting, rearing and governing children until they are twenty-one years of age, with a view of ascertaining to what degree of physical excellence the human race may attain under the most favorable conditions of healthful environment during the formational and developmental period of life. There doesn't seem to be, among all the strivings after perfection in every other department of life, any desire, any curiosity, even to know what a perfect physical being man might become under circumstances which are in consonance with nature in his begetting, in his rearing and in his moral, mental and physical endowment.

It looks as though something had discouraged the race from attempting anything of the kind. What is this something? Can it be possible that revealed religion has had something to do with this lack of endeavor toward the grand physical climacteric that has been intimated? Is there not some good ground for believing that that which is regarded as the chiefest boon of the race, spiritually, has resulted in its physical degeneracy? Is the doctrine of total depravity to be held responsible for all this absence of endeavor toward physical integrity? Are we not discouraged before entering upon such a work by the too literal acceptance of the "prone to evil" dogma, of the "born in sin" pronunciamento, of the "total depravity" principle? Aren't we too willing to accept the theory that all the waywardness is from within outward, rather than from without inward? Isn't it regarded as something sacrilegious to even question the too credulously accepted dicta of the presumptious? And would not the so-called orthodoxy of the universe cry out against the initial effort, nay, even against the suggestion of such endeavor?

It looks like it. May be, in the coming time, there will arise one who may hit upon a formula that may seem not to defy established dogma, and reconcile it with the grand endeavor to test the physical possibilities of our race, with a view to developing perfect specimens of the genus homo. It may not, cannot be done in one generation—perhaps not in a score—and it may take hundreds; but isn't it worth trying for? For if so be that man has lived all these centuries in a comparatively enfeebled, phyically degenerate state, in no sense the perfect being his Creator intended, lo, the grand pity! Alas for the loss all these ages through!

We hear much about the strife among rearers of horses and cattle and fowls and fruit and vegetables, to breed and cultivate and develop toward perfection; but we do not hear of any effort to attain to the grand possibilities of the human soul, mind and body. Suppose that for, say ten generations, selected specimens of the best of the race were colonized and reared under the best system of physical, mental and moral development that man can conceive of, apart from all contact with the degenerating tendencies of our so-called civilization; isn't the probable result something to yearn after, something worth striving for? The results of such a culture might—doubtless would—revolutionize the methods of human living ever after, and produce a race of which it would not seem sacrilegious to say that it is created in the image of God, and after His likeness.

